# Natural killer cells and natural killer T cells in Lyme arthritis

**DOI:** 10.1186/ar4373

**Published:** 2013-11-07

**Authors:** Kia Katchar, Elise E Drouin, Allen C Steere

**Affiliations:** 1 Center for Immunology and Inflammatory Diseases, Division of Rheumatology, Allergy and Immunology, Massachusetts General Hospital, Harvard Medical School, CNY 149/8301, 55 Fruit St, Boston, MA 02114, USA; 2 Current address for K Katchar is Vertex Pharmaceuticals, 130 Waverly Street, Cambridge, MA 02139, USA

## Abstract

**Introduction:**

Natural killer (NK) and natural killer T (NKT) cells provide a first line of defense against infection. However, these cells have not yet been examined in patients with Lyme arthritis, a late disease manifestation. Lyme arthritis usually resolves with antibiotic treatment. However, some patients have persistent arthritis after spirochetal killing, which may result from excessive inflammation, immune dysregulation and infection-induced autoimmunity.

**Methods:**

We determined the frequencies and phenotypes of NK cells and invariant NKT (iNKT) cells in paired peripheral blood (PB) and synovial fluid (SF) samples from eight patients with antibiotic-responsive arthritis and fifteen patients with antibiotic-refractory arthritis using flow cytometry and cytokine analyses.

**Results:**

In antibiotic-responsive patients, who were seen during active infection, high frequencies of CD56bright NK cells were found in SF, the inflammatory site, compared with PB (*P* <0.001); at both sites, a high percentage of cells expressed the activation receptor NKG2D and the chaperone CD94, a low percentage expressed inhibitory killer immunoglobulin-like receptors (KIR), and a high percentage produced IFN-γ. In antibiotic-refractory patients, who were usually evaluated near the conclusion of antibiotics when few if any live spirochetes remained, the phenotype of CD56bright cells in SF was similar to that in patients with antibiotic-responsive arthritis, but the frequency of these cells was significantly less (*P* = 0.05), and the frequencies of CD56dim NK cells tended to be higher. However, unlike typical NKdim cells, these cells produced large amounts of IFN-γ, suggesting that they were not serving a cytotoxic function. Lastly, iNKT cell frequencies in the SF of antibiotic-responsive patients were significantly greater compared with that of antibiotic-refractory patients where these cells were often absent (*P* = 0.003).

**Conclusions:**

In patients with antibiotic-responsive arthritis, the high percentage of activated, IFN-γ-producing CD56bright NK cells in SF and the presence of iNKT cells suggest that these cells still have a role in spirochetal killing late in the illness. In patients with antibiotic-refractory arthritis, the frequencies of IFN-γ-producing CD56bright and CD56dim NK cells remained high in SF, even after spirochetal killing, suggesting that these cells contribute to excessive inflammation and immune dysregulation in joints, and iNKT cells, which may have immunomodulatory effects, were often absent.

## Introduction

Lyme disease, which occurs in North America, Europe and Asia, is caused by infection with tick-borne spirochete *Borrelia burgdorferi*[1]. Lyme arthritis, a late manifestation of the disorder [2], can usually be treated successfully with one month of oral or intravenous (IV) antibiotics, termed antibiotic-responsive arthritis [3,4]. However, in a small percentage of patients, proliferative synovitis persists for months to several years despite two to three months of oral and IV antibiotics, called antibiotic-refractory arthritis [5]. This complication has been noted primarily in patients with Lyme arthritis in the northeastern United States.

Antibiotic-refractory Lyme arthritis is more frequent in patients infected with highly inflammatory RNA intergenic spacer type 1 (RST1) strains of *B. burgdorferi*[6], which account for 30 to 50% of the infections in the northeastern U.S. [7,8]. However, culture and PCR results for *B. burgdorferi* in synovial tissue obtained at synovectomy during the post-antibiotic period have been uniformly negative [9], suggesting that refractory arthritis does not result from persistent infection. Rather, in genetically susceptible individuals, particularly those with a Toll-like receptor 1 (TLR1) polymorphism [10] and/or certain HLA-DR alleles [11], excessive joint inflammation associated with certain *B. burgdorferi* strains [6], *B. burgdorferi* proteins [12] or spirochetal antigens adherent to cartilage surfaces [13], may lead to immune dysregulation of CD4+ T cell subsets [14,15] and infection-induced autoimmunity [16], resulting in persistent joint inflammation after spirochetal killing with antibiotic therapy.

T cells, B cells, and natural killer (NK) cells make up the three lineages of lymphocytes. NK cells, which comprise 5 to 12% of peripheral blood (PB), provide a first line of defense against infection, and they can spontaneously kill virally infected or malignant cells [17,18]. More recently, it has been shown that NK cells also have immunoregulatory properties that influence the adaptive immune response, and maladaption of these responses may lead to autoimmunity [19,20]. The functional activity of NK cells is regulated through their repertoire of activating and inhibitory receptors that recognize ligands on the surface of cells.

NK cells can be subtyped based upon their expression levels of CD56, a homophilic neural cell adhesion molecule. The majority of NK cells in PB are CD56dim cells, whereas CD56bright cells are typically increased at sites of inflammation [18]. CD56bright NK cells usually have low or negligible expression of the FcγRIII receptor (CD16low/-); they are typically noncytotoxic, and they generally express high levels of cytokines [19]. In contrast, CD56dim NK cells, which are thought to be derived from CD56bright cells, are usually CD16high; they are typically cytotoxic, and they express low levels of cytokines [21]. However, depending upon the inflammatory milieu, the phenotype and function of either NK cell subtype can be changed leading to altered effector or regulatory functions [18].

Natural killer T (NKT) cells, a subgroup of T cells comprising about 0.2% of lymphocytes, express surface antigens associated with both adaptive (T cell receptor) and innate (NK receptors) immunity [22]. NKT cells, which are potent producers of cytokines, foster protective immunity to microbes and tumor cells, but also help maintain tolerance to self. In humans, NKT cells recognize lipids presented by a family of nonclassical MHC class I molecules called CD1a, b, c, d, or e [23]. Because of this variability, NKT cells are classified based upon their recognition of specific CD1 molecules and expression of specific α and β chains of the T cell receptor. The most studied NKT cell subtype is invariant NKT (iNKT) cells, the only type found in mice. In humans, the iNKT cell receptor expresses the Vα24-Jα18 α chain and the Vβ11 β chain [24].

In Lyme disease, NK and NKT cells have been assessed in tissue culture [25], in murine models of *B. burgdorferi* infection [25-27], and in patients with erythema migrans (EM), an early disease manifestation [28]. However, it is not yet known whether NK or NKT cells play a role late in the illness in patients with Lyme arthritis. We report here that patients with antibiotic-responsive arthritis had high percentages of activated IFN-γ-producing CD56bright NK cells in synovial fluid (SF) and increased frequencies of iNKT cells there, suggesting that these cells still have a role in spirochetal killing late in the illness. However, in patients with antibiotic-refractory arthritis, the frequencies of IFN-γ-producing CD56bright and CD56dim NK cells remained high in SF after spirochetal killing, suggesting that these cells contribute to excessive inflammation and immune dysregulation in joints, and iNKT cells, which may have immunomodulatory effects, were often absent.

## Methods

### Study population

Paired samples of PB and SF from twenty-three patients with Lyme arthritis, eight with antibiotic-responsive arthritis and fifteen with antibiotic-refractory arthritis, were available for most experiments except where indicated. The Human Investigation Committee at Massachusetts General Hospital (MGH) approved the study, entitled ‘Immunity in Lyme arthritis’, and all patients (or the parents of patients who were minors) provided written informed consent. All patients met the Center for Disease Control and Prevention criteria for the diagnosis of Lyme disease [29], and received antibiotic therapy according to the guidelines of the Infectious Diseases Society of America [30]. For comparison, PB was also obtained from four healthy control subjects.

As in the past [5], antibiotic-responsive arthritis was defined as resolution of arthritis within three months after four to eight weeks of oral antibiotics or ≤4 weeks of IV antibiotics. Antibiotic-refractory arthritis was defined as persistent joint swelling for >3 months after ≥8 weeks of oral antibiotics and ≥4 weeks of IV antibiotics. PCR and antibody testing for *B. burgdorferi* were determined as previously described [9], and the total white cell counts and the percentages of polymorphonuclear leukocytes in SF were measured in the MGH clinical laboratories.

### Surface staining of cells and flow cytometry

For enumeration of the various immune cell populations, PB and SF cells were thawed and washed in phosphate-buffered saline (PBS) in the presence of 1% fetal calf serum and incubated for 30 minutes at 4°C. Lymphocytes were identified by their forward and side scatter characteristics. NK cells were distinguished based upon CD56 expression (PerCp Cy5.5 anti-human CD56) within the gated CD3-negative (FITC anti-human CD3) lymphocyte population. To discriminate between the CD56bright (high intensity) and CD56dim (low intensity) NK cell subpopulations, two regions were established within the CD3-CD56+ NK cell gate in accordance with previous reports [31,32]. Invariant NKT cells were distinguished based upon CD56 and Vα24 expression within the CD3-positive lymphocyte gate. All samples for this panel were analyzed on FACSCalibur using CellQuest software (Becton Dickinson, Franklin Lakes, NJ, USA).

To determine inhibitory and activating receptors expression on NK cells, the monoclonal antibody (mAb) panel consisted of phycoerythrin (PE) anti-human killer immunoglobulin-like receptors (KIRs) (CD158e1 KIR3DL1 (DX9)) and CD158b KIR2DL2/2DL3 (DX27), PerCp Cy5.5 anti-human CD56, Pacific Blue anti-human CD3, Alexa Fluor™ 700 anti-human CD16, Alexa Fluor™ 647 anti-human CD94 (DX22), and PE/Cy7 anti-human CD314 (NKG2D). The samples were analyzed using a four-laser LSRII (Becton Dickinson) and FlowJo software (TreeStar, Ashland, OR, USA).

### *B. burgdorferi* culture and enumeration

A low-passage *B. burgdorferi* RST1 strain (EM70), a strain type with greater inflammatory potential than other strains [33], was cultured in Barbour-Stoenner-Kelly (BSK) medium containing 6% rabbit serum (Sigma-Aldrich, St Louis, MO, USA) at 37°C with 5% CO_2_. Spirochetes were harvested at late-log phase (0.5 to 2 × 10^9^) by centrifugation at 14,000 rpm, washed twice with PBS, and resuspended in RPMI medium. The number of organisms was enumerated by dark-field microscopy using a Petroff-Hausser counting chamber.

### Cell stimulation and intracellular staining for cytokines

For cytokine analyses, cells were thawed and resuspended in culture media (RPMI 1640, supplemented with 10% fetal calf serum, 1% penicillin/streptomycin and 2 mM L-glutamine); they were counted and plated in 96-well round bottom plate at a concentration of 2 × 10^5^ per well. Cells were stimulated with *B. burgdorferi* at a MOI of 25:1 for 24 h at 37°C and 5% CO_2_. As controls, cells were stimulated either with phorbol 12-myristate-13-acetate (10 ng/ml) and ionomycin (0.5 μg/ml) (positive control) or media alone (negative control) (data not shown). To inhibit cytokine secretion, 2 μM monensin (GolgiStop; BD Biosciences Pharmingen, San Diego, CA, USA) was added for the last 6 h.

Cells were harvested and stained for the surface marker CD56 and CD3 at 4°C for 20 min. After incubation, the cells were washed twice with PBS and intracellular staining was performed using a Cytofix/Cytoperm™ kit (BD Biosciences) following the manufacturer’s instructions. Cells were stained with Alexa Fluor™ 488 anti-human IFN-γ, PE-Cy7 anti-human TNF-α and PerCp Cy5.5 anti-human IL-4 mAbs, fixed with 4% paraformaldehyde and analyzed by flow cytometry.

### Statistical analysis

The distribution of values between groups was compared by Mann-Whitney rank sum test. The Prism statistical program (GraphPad Software, San Diego, CA, USA) was used for all analyses. All *P* values were two-tailed, and *P* values <0.05 were considered statistically significant.

## Results

### Clinical characteristics of patients

The demographic and clinical characteristics of the twenty-three study patients with Lyme arthritis and four healthy control subjects are shown in Table 1. Among the eight patients with antibiotic-responsive arthritis, SF samples were usually obtained prior to or soon after the initiation of antibiotic treatment when the infection was still active. At that time, PCR results for *B. burgdorferi* DNA in SF were positive in all patients, and the median white cell count in SF was 25,750 cells/mm^3^. These patients received oral doxycycline for one or two months, in two cases followed by IV ceftriaxone for one month, and by definition, their arthritis resolved within three months after the start of antibiotics.

**Table 1:** Clinical characteristics of the twenty-three patients with Lyme arthritis and of four healthy control subjects*

	**Responsive**	**Refractory**	***P *** **value**
	N = 8**	N = 15**	
Age, years	44 (17-53)	29 (12-78)	0.2
No. male/female	5/3	8/7	0.6
Duration of arthritis (months)			
Before treatment	0.1 (0.1-1)	1 (0.1-6)	0.06
From start of antibiotics to sample date	0 (0-3)	4 (0-18)	0.001
From start of antibiotics to resolution of arthritis	3 (1.5-3)	10 (4-20)	0.0001
Serum findings on the sample date			
Positive serologic result for *B. burgdorferi*			
antibodies by ELISA and Western blotting	8	15	1.0
Joint fluid findings on the sample date			
Total white cell count/mm^3^	25,750	12,150	0.01
	(13,833-60,000)	(5,694-46,250)	
% polymorphonuclear leukocytes	79 (28-91)	88 (16-98)	0.35
Total mononuclear cells/mm3	1,600	1,500	0.69
	(400-6,500)	(320-6,300)	
No. PCR+/PCR- for *B. burgdorferi* DNA	8/0	5/10	0.002

*The four healthy control subjects had a median age of 36 (range, 28 to 42; three were women and one was a man. None had a positive serologic test result for *B. burgdorferi* antibodies.

**All values are given as median (range) unless otherwise noted. ELISA, enzyme-linked immunosorbent assay.

In contrast, the fifteen patients with antibiotic-refractory arthritis included one who was referred to our clinic before antibiotic therapy, five who were referred after one to two months of oral antibiotics, and nine who were referred at the completion of two months of oral antibiotics and one month of IV antibiotic treatment. By definition, all fifteen patients received one to two months of oral antibiotics and one month of IV antibiotics, but their arthritis did not resolve within three months after the start of this therapy. The PB and SF samples from patients with refractory arthritis were obtained when the patients were first seen in our clinic, a median duration of four months (range zero to eighteen months) after the start of antibiotics (Table 1). On the sample date, only five of the fifteen patients had positive PCR results for *B. burgdorferi* DNA in SF, and the median total white cell count in SF was 12,150 cells/mm^3^. These values, which were usually obtained when few, if any, live spirochetes remained, were significantly less than those in patients with antibiotic-responsive arthritis (Table 1). However, in both the refractory and responsive groups, most of the white cells in SF were polymorphonuclear leukocytes (median value, 88% versus 79%).

After the completion of antibiotic therapy, the patients with antibiotic-refractory arthritis were treated with disease-modifying anti-rheumatic drugs (DMARDs) followed in one case by arthroscopic synovectomy. Their arthritis resolved a median duration of ten months (range, four to twenty months) after the start of antibiotics.

### Frequencies of NK cell subsets

**Figure 1:**
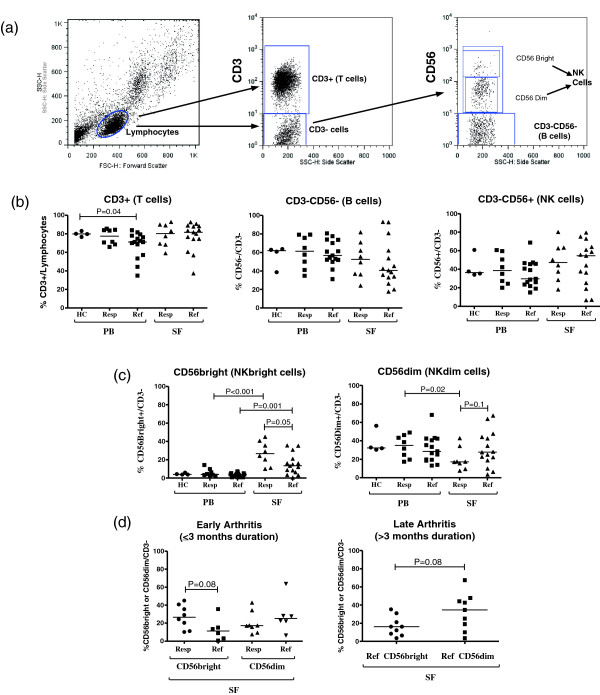
**Frequencies of various immune cell subsets in patients with antibiotic-responsive or antibiotic-refractory Lyme arthritis. (a)** Representative dot plots of peripheral blood (PB) from one patient showing the gating strategy for the various lymphocyte populations. **(b and c)** Enumeration of various immune cell subsets of matched PB and synovial fluid (SF) samples from eight antibiotic-responsive (Resp) and fifteen antibiotic-refractory (Ref) patients, and for comparison four healthy control (HC) subjects. **(d)** Frequencies of cells in the SF of patients with antibiotic-refractory arthritis were categorized based upon whether the sample was obtained early (≤3 months, N = 6) or late (>3 months, N = 9) after the start of antibiotics. The graph on the left shows the percentages of CD56bright and CD56dim natural killer (NK) cells in refractory patients whose samples were collected early in the course of their arthritis (≤3 months) compared with those from responsive patients. The graph on the right compares the frequencies of CD56bright and CD56dim NK cells in refractory patients whose samples were obtained >3 months after the start of antibiotics. Symbols indicate individual subjects, horizontal bars represent the median of each group and significant differences are denoted. Statistical analyses were performed by using the Mann-Whitney test.

Although the total white cell counts in SF were significantly greater in patients with antibiotic-responsive arthritis than in those with antibiotic-refractory arthritis, the frequencies of mononuclear cells at that site were similar in the two patient groups (Table 1). The gating strategy to distinguish the various lymphocyte populations within the pool of mononuclear cells is shown in Figure 1a. When gated on lymphocytes, PB from healthy control subjects and PB and SF from patients with antibiotic-responsive or antibiotic-refractory arthritis had similar percentages of CD3+ T cells, CD3-CD56+ NK cells, and CD3-CD56- cells, which were primarily B cells (Figure 1b).

When NK cells were subtyped based on CD56 expression levels, the median frequencies of CD56bright NK cells were significantly greater in SF than PB in both patient groups (*P* ≤0.001). However, their frequencies were significantly greater in the SF of patients with antibiotic-responsive arthritis compared with those with antibiotic-refractory arthritis (median values, 27% versus 13%, *P* = 0.05) (Figure 1c). In contrast, the median frequency of CD56dim NK cells was significantly lower in SF than PB in patients with antibiotic-responsive arthritis (*P* = 0.02). However in patients with antibiotic-refractory arthritis, the median frequency of these cells was similar in SF and PB. Consequently, the frequency of CD56dim NK cells in SF tended to be greater in patients with refractory arthritis than in those with responsive arthritis (median value, 28% versus 17%, *P* = 0.1).

Since CD56bright NK cells may differentiate into CD56dim NK cells over time, the frequencies of these subsets in SF in patients with antibiotic-refractory arthritis were assessed according to the duration of arthritis after the start of antibiotics. Only one patient with refractory arthritis was evaluated prior to antibiotic therapy, the time point when most patients with responsive arthritis were seen, and the percentage of CD56bright NK cells in that patient (0.6%) was very low. In addition, five other patients were seen prior to the completion of oral and IV antibiotic therapy (≤3 months after the initiation of antibiotics) when some live spirochetes may have been present, and nine were evaluated after the completion of antibiotics (>3 months after the start of antibiotics) when few, if any, live spirochetes remained.

Among the six patients with refractory arthritis who were seen early in the course of the arthritis prior to the completion of antibiotics (Figure 1d), the frequencies of CD56bright NK cells tended to be less in those with refractory arthritis than in those with responsive arthritis (*P* = 0.08), whereas the frequencies of CD56dim NK cells were slightly greater in the refractory group. Similarly, among the nine patients in the refractory group who were seen late in the course of the arthritis after the completion of antibiotics, CD56bright NK cells again tended to be less frequent than CD56dim NK cells (*P* = 0.08) (Figure 1d). Thus, regardless of when the sample was obtained, CD56dim NK cells were often predominant in the SF of patients with antibiotic-refractory arthritis and the frequencies of CD56bright NK cells were usually lower.

Since age and sex may influence the frequencies of NK cells, the patients with antibiotic-responsive or antibiotic-refractory arthritis were each stratified into two groups according to age (those above or below the median value) and sex (men or women). However, no significant differences were noted in cell frequencies according to these variables (data not shown).

### Receptor expression of NK cell subsets

The expression of activation or inhibitory receptors was assessed on CD56bright NK cells and CD56dim NK cells in PB and SF from four or five patients each with responsive or refractory arthritis in whom enough cells were available. As with the larger group of 23 patients, the smaller group with antibiotic-responsive arthritis were evaluated prior to or soon after the start of antibiotic therapy, and those with antibiotic-refractory arthritis were seen at the completion of oral or IV antibiotic therapy.

**Figure 2:**
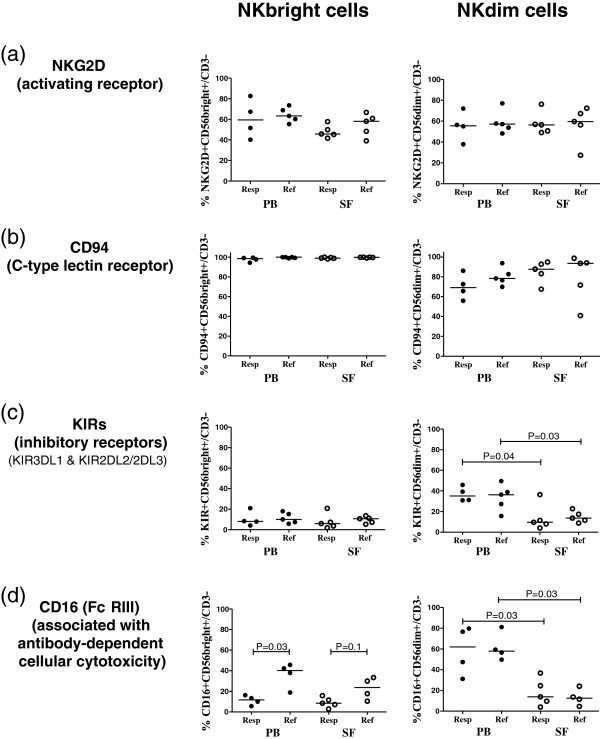
**Frequencies of CD56bright and CD56dim NK cells that express the receptors NKG2D, CD94, KIRs (KIR3DL1 and KIR2DL2/2DL3) or CD16. (a-b)** Peripheral blood (PB) and synovial fluid (SF) samples from four to five patients with antibiotic-responsive arthritis and four to five patients with antibiotic-refractory arthritis were analyzed for expression of NKG2D **(a)**; CD94 **(b)**; killer immunoglobulin-like receptors (KIRs) **(c)**; or CD16 **(d)**. For three of the five responsive patients, PB and SF samples were matched, and all PB and SF samples from refractory patients were matched. Symbols indicate individual subjects, horizontal bars represent the median of each group and significant differences are denoted. Statistical analyses were performed by using the Mann-Whitney test.

In both patient groups, high frequencies of CD56bright and CD56dim NK cells in PB and SF expressed the activating receptor NKG2D and the chaperone molecule CD94, which can pair with either activating or inhibiting receptors (Figures 2a and b). In contrast, low frequencies of CD56bright NK cells in PB and SF of both patient groups expressed the inhibitory KIRs, which repress the activity of NK cells (Figure 2c). However, the frequencies of CD56dim NK cells that expressed KIR receptors were significantly lower in SF compared with PB of both patients with responsive arthritis (*P* = 0.04) and in those with refractory arthritis (*P* = 0.03), suggesting that CD56dim NK cells were less inhibited in SF than PB.

Expression of the FcγRIII receptor (CD16), which is associated with antibody-dependent cellular cytotoxicity (ADCC), is usually low or negligible on CD56bright NK cells, but high on CD56dim NK cells, which typically serve a cytotoxic function [19]. Here, this usual pattern of CD16 expression was observed in PB from patients with antibiotic-responsive arthritis (Figure 2d). However, in both patient groups, CD16 expression on CD56dim NK cells was significantly lower in SF than PB (*P* = 0.03). Moreover, in patients with refractory arthritis, the usual pattern of CD16 expression tended to be reversed in SF. At that site, the refractory group tended to have higher expression of CD16 on CD56bright NK cells (median value = 24%) compared with responsive patients (median value = 8%), whereas CD16 expression in CD56dim NK cells was relatively low in the SF of both patient groups. Thus, the low expression of CD16 on CD56dim NK cells in SF suggested that these cells were not serving a cytotoxic function in SF in patients with responsive or refractory Lyme arthritis.

### Cytokine profile of *B. burgdorferi*-stimulated NK cells

Following stimulation with *B. burgdorferi* for 24 h, intracellular cytokine production by NK cells was determined in the SF of six patients with antibiotic-responsive arthritis and seven patients with antibiotic-refractory arthritis in whom enough cells were available. In these patients, the timing of when the samples were obtained was representative of the larger group of 23 patients.

**Figure 3:**
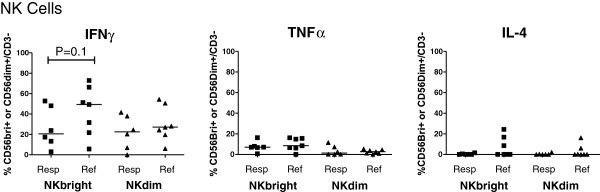
**Frequencies of SF CD56bright and CD56dim NK cells that secrete IFNγ, TNFα and IL-4 in patients with antibiotic-responsive or antibiotic-refractory arthritis.** Synovial fluid (SF) cells from six antibiotic-responsive (Resp) and seven antibiotic-refractory (Ref) patients were stimulated with *B. burgdorferi* at a multiplicity of infection (MOI) of 25:1 for 24 h. For the last 6 h, GolgiStop was added and the presence of IFNγ, TNFα and IL-4 was measured by intracytoplasmic staining and flow cytometry. Symbols indicate individual subjects and horizontal bars represent the median of each group. Statistical analyses were performed by using the Mann-Whitney test.

The frequency of CD56bright NK cells that produced IFN-γ tended to be higher in the refractory group than in the responsive group (median 49% versus 21%, *P* = 0.1) (Figure 3), and the frequencies of CD56dim NK cells that produced this cytokine were also substantial in both the responsive and refractory groups (approximately 20%), suggesting that both cell types were often polarized to a Th1 phenotype. In addition, in both patient groups, approximately 5% of CD56bright NK cells produced TNF-α, but only a few CD56dim NK cells produced this cytokine. A few patients in the refractory group had low percentages of IL-4 staining cells, but in most patients, such cells were not detectable. Thus, in patients with Lyme arthritis, both CD56bright and CD56dim NK cells appear to secrete primarily the Th1 cytokine IFN-γ, and in patients with antibiotic-refractory arthritis, both CD56bright and CD56dim NK cells continue to do so in the post-antibiotic period.

### Frequencies of invariant NKT cells

**Figure 4:**
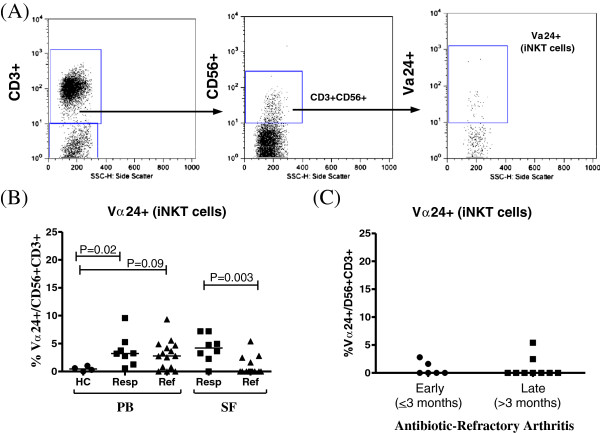
**Frequencies of Vα24+ (invariant) NKT cells in patients with antibiotic-responsive or antibiotic-refractory arthritis. (a)** Representative dot plots of peripheral blood (PB) from one patient showing the gating strategy used to determine the frequency of invariant natural killer T cells (iNKT) in patient samples. **(b)** Frequencies of iNKT cells in matched PB and synovial fluid (SF) samples from eight antibiotic-responsive (Resp) and fifteen antibiotic-refractory (Ref) patients, and for comparison four healthy control (HC) subjects. **(c)** Frequencies of iNKT cells in the SF of patients with antibiotic-refractory arthritis stratified by whether the sample was obtained early (≤3 months, N = 6) or late (>3 months, N = 9) after the start of antibiotics. Symbols indicate individual subjects, horizontal bars represent the median of each group and significant differences are denoted. Statistical analyses were performed by using the Mann-Whitney test.

The frequencies of iNKT cells (CD3 + CD56 + Vα24+) were determined in all 23 patients with antibiotic-responsive or antibiotic-refractory arthritis, and in healthy control subjects. The gating strategy used to identify these cells is shown in Figure 4a. Compared with healthy subjects, the median frequencies of iNKT cells were increased in PB in both patient groups (median values, 0.5% versus approximately 3%) (Figure 4b). In SF, the frequencies of iNKT in patients with antibiotic-responsive arthritis were similar to that in PB. However, in patients with antibiotic-refractory arthritis, iNKT cells were usually undetectable in SF. Overall, seven of eight patients with responsive arthritis had detectable iNKT cells in SF compared with only four of fifteen patients with refractory arthritis (*P* = 0.003).

When the frequencies of iNKT cells in patients with refractory arthritis were stratified according to when the cells were obtained, similar frequencies were found in patients who were seen prior to the completion of antibiotics (≤3 months, N = 6) and in those seen after the completion of antibiotics (>3 months, N = 9) (Figure 4c). Similarly, stratification of iNKT cell frequencies by age and sex did not show significant differences (data not shown). Because iNKT cells were often absent in SF in the refractory group, the cytokine profiles of these cells were not assessed.

## Discussion

In this study, we compared NK and iNKT cell frequencies and phenotypes in PB and SF in patients with antibiotic-responsive or antibiotic-refractory arthritis. In other forms of chronic inflammatory arthritis, it has been necessary to compare findings in patients to those in patients with another disease or healthy control subjects. However, one can only obtain PB and not SF from healthy subjects. In Lyme arthritis, in which the arthritis is often localized to one knee, the frequencies and phenotypes of immune cells in PB have often been similar in patients and healthy control subjects [14,15], as was the case here. Instead, the most informative comparisons have been of SF obtained from antibiotic-responsive patients during infection versus SF obtained from antibiotic-refractory patients during the post-antibiotic period when excessive inflammation, immune dysregulation, and infection-induced autoimmunity are thought to play a pathogenetic role in the illness [15,16].

In the current study, patients with antibiotic-responsive arthritis, who were evaluated during active infection, had high frequencies of CD56bright NK cells in SF, the site of inflammation. In addition, a high percentage of the cells expressed the activation receptor NKG2D and the chaperone CD94, whereas a low percentage expressed inhibitory KIR receptors. Furthermore, a high percentage of the cells produced IFN-γ, a characteristic Th1-associated cytokine that is prominent during infection [34-36]. This is the typical phenotype of CD56bright NK cells at sites of inflammation [18], and suggests that in Lyme disease, these cells still have a role in control of the infection in joints late in the illness.

In comparison, patients with antibiotic-refractory arthritis, who were usually seen near or at the conclusion of antibiotic therapy when few, if any, live spirochetes remained [10], had a similar activated phenotype of CD56bright cells in SF as in patients with antibiotic-responsive arthritis, but the frequencies of these cells were significantly less. Instead, patients with antibiotic-refractory arthritis tended to have more CD56dim NK cells in SF. Since CD56bright NK cells may evolve into CD56dim cells as they mature, we considered whether this finding could be explained simply by the fact that the cells in antibiotic-refractory patients were usually obtained during the post-antibiotic period. However, even in refractory patients in whom the cells were collected earlier in the course of the arthritis, the percentages of CD56bright cells were less and the numbers of NKdim cells proportionally more than in patients with responsive arthritis, suggesting that the differences between the two patient groups were not simply a function of time.

Rather than the typical phenotype of CD56dim cells, these cells in both responsive and refractory patients often produced IFN-γ, and had low expression of CD16 receptors, suggesting that the cells were not serving their usual cytotoxic function [23]. Rather, the frequencies of IFN-γ-producing CD56bright and CD56dim remained high, even after spirochetal killing. We have previously shown that patients with antibiotic-refractory arthritis have significantly higher levels of proinflammatory cytokines and chemokines in SF, particularly IFN-γ and the IFN-inducible chemokines CXCL9 and CXCL10, than patients with antibiotic-responsive arthritis [10,37]. Moreover, this highly inflammatory milieu is associated with a persistent imbalance of the CD4+ effector T/regulatory T cell ratio in SF weighted toward effector T cells, such that the higher the ratio the longer the post-antibiotic duration of arthritis [15]. Thus, in refractory patients, IFN-γ-producing CD56bright and CD56dim NK cells may contribute to the excessive inflammation and immune dysregulation that is characteristic of antibiotic-refractory Lyme arthritis.

As with NK cells, patients with antibiotic-responsive arthritis had higher frequencies of iNKT cells in SF than PB, but these cells were often absent in SF in patients with antibiotic-refractory arthritis. The first demonstration of a glycoplipid antigen from a human pathogen presented by CD1d molecules to iNKT cells was *B. burgdorferi* monogalactosyl diacylglycerol (*Bb*GLII) [25], one of the two *B. burgdorferi* glycolipids [38,39]. Furthermore, in mice, which only express CD1d molecules, iNKT cells were shown to be important for control of early infection [25-27]. In humans, skin explants from healthy control subjects had constitutive expression of CD1a, but low expression of CD1b and CD1c molecules [28]. When the explants were cultured with *B. burgdorferi*, CD1b and CD1c expression increased substantially on myeloid dendritic cells. Finally, both acylated cholesteryl galactoside (*Bb*GLI) and *Bb*GLII induce robust antibody responses in approximately one-third of patients with early infection and in almost all patients with Lyme arthritis, including those with refractory arthritis [39]. Thus, iNKT cells in SF likely help B cells to produce antibodies to *B. burgdorferi* glycolipids, which may aid in spirochetal killing. However, the absence of these cells in SF in patients with refractory arthritis may contribute to the inability of these patients to downmodulate their immune response after spirochetal clearance. Furthermore, the low level or lack of these cells in SF suggests that self lipids do not play a role in autoimmunity in the post-antibiotic period in patients with antibiotic-refractory arthritis.

In autoimmune diseases, it has been suggested that CD56bright NK cells may either exacerbate or regulate immune responses [40]. In rheumatoid arthritis (RA), CD56bright NK cells are greatly enriched in SF or synovial tissue, and may engage with monocytes in a reciprocal program of activation [41]. In comparison, the frequency of these cells in PB may be less than in healthy control subjects [42], presumably because the cells have migrated to inflamed joints. In patients with multiple sclerosis, clinical remission after treatment with IFN-β and daclimumab was associated with an expansion of CD56bright NK cells in PB [43], but this may result from the exiting of these cells from sites of inflammation.

In RA, it has also been questioned whether iNKT cells are contributors or modulators of inflammation [40]. In RA patients, clinical improvement in response to rituximab was associated with increased numbers of iNKT cells in PB [44], but again, this could be caused by the cells leaving inflamed joints. On the other hand, in murine collagen-induced arthritis, iNKT cell secretion of IFN-γ suppressed Th17 cell differentiation; it induced regulatory T cells, and it caused apoptosis of autoreactive T cells [45]. Similarly, in KBxN mice, iNKT cells activated by α-galactosylceramide (α-GalCer) suppressed glucose-6-phosphate isomerase (GPI) peptide-induced arthritis by suppression of GPI-specific CD4+ T cells [46]. In future experiments, it will be important to explore both the effector and immunoregulatory potential of these cells in patients with Lyme arthritis.

It has been reported that determination of NK cell numbers, identified by CD57, a marker for replicative senescence, may provide evidence for activity of disease and response to therapy in patients with post-Lyme disease pain, neurocognitive and fatigue symptoms [47]. However, using both CD56 and CD57 staining, others found that NK cell counts were similar in patients with post-Lyme symptoms and in healthy control subjects [48]. Although we noted differences in the frequencies of NK cell subsets in SF compared with PB, NK cell numbers were similar in PB in patients with either responsive or refractory Lyme arthritis and in healthy control subjects.

## Conclusions

In patients with antibiotic-responsive arthritis, who were seen during active infection, the high percentages of activated, IFN-γ-producing CD56bright NK cells in SF and the presence of iNKT cells suggest that these cells still have a role in spirochetal killing late in the illness. In patients with antibiotic-refractory arthritis, the frequencies of IFN-γ-producing CD56bright and CD56dim NK cells in SF remained high, even after spirochetal killing, suggesting that these cells contribute to excessive inflammation and immune dysregulation in joints. Additionally, the lack of iNKT cells in the SF of refractory patients suggests that their absence may further contribute to the immune dysregulation experienced by these patients. Overall, quantitative rather than qualitative differences in NK and iNKT cell populations appear to contribute to an antibiotic-refractory arthritis outcome.

## Abbreviations

ADCC: Antibody-dependent cellular toxicity; B. burgdorferi: *Borrelia burgdorferi*; BbGLI: *Borrelia burgdorferi* glycolipid I; BbGLII: *Borrelia burgdorferi* glycolipid II; EM: Erythema migrans; GPI: Glucose-6-phosphate isomerase; IFN: Interferon; IL: Interleukin; IV: Intravenous; KIR: Killer immunoglobulin-like receptor; MGH: Massachusetts General Hospital; NK: Natural killer cells; iNKT: Invariant natural killer T cells; mAb: Monoclonal antibody; PB: Peripheral blood; PBS: Phosphate-buffered saline; PE: Phycoerythrin; RA: Rheumatoid arthritis; RST: RNA intergenic spacer type; SF: Synovial fluid; TLR: Toll-like receptor; TNFα: Tumor necrosis factor alpha.

## Competing interests

Dr. Steere serves as a consultant to Baxter Bioscience; fees < $10,000. Drs. Katchar and Drouin declare that they have no competing interests.

## Authors’ contributions

KK, EED and ACS had full access to all of the data in the study and share responsibility for the integrity of the data and the accuracy of data analysis. KK was responsible for the acquisition of the data; KK and ACS were responsible for the design of the study, and all three authors were involved in the preparation of the manuscript, and performed statistical analyses. All authors read and approved the final manuscript.
